# Algal sensitivity to nickel toxicity in response to phosphorus starvation

**DOI:** 10.1038/s41598-022-25329-5

**Published:** 2022-12-05

**Authors:** Nagwa I. El-Agawany, Mona I. A. Kaamoush

**Affiliations:** 1 Botany and Microbiology Department, Faculty of Science, Alexandria University, Alexandria, Egypt; 2 Environmental Protection and Crises Management Department, Simulator Complex, Arab Academy for Science, Technology and Maritime Transport (AAST), Alexandria, Egypt

**Keywords:** Biological techniques, Ecology

## Abstract

In all living cells, phosphorus plays an important function in the transport of metabolic energy and as a structural component of nucleotide and phospholipid molecules. Ni^2+^ is found in industrial water effluent and has the potential to harm aquatic ecosystems. The study was designed to assess the effect of phosphorous-limiting conditions in combination with the absence or presence of different concentrations of nickel on growth, pigment content, photosynthesis, and respiration activities of the studied alga *Dunaliella tertiolecta*. The EC_50_ for dissolved nickel was around 15 mg/L. Results obtained showed that, growth and chlorophyll content under phosphorus limiting conditions increased under low concentrations of dissolved nickel. The amount of O_2_-evolution with phosphorus limiting conditions was lower than those of untreated normal cultures. Lower dissolved nickel concentrations resulted in higher photosynthesis rates in the treated phosphorus-starved cultures than higher concentrations. The degree of response to metal toxicity in phosphorus-starved medium is depending mainly on the concentration of the element and the length of the culturing period and it was less than those in normal control culture containing phosphorus.

## Introduction

Phosphate is often the limiting nutrient in many ecosystems, despite its abundance in nature, because it is mostly found in forms that aren’t readily available, such as insoluble salts^1^. Nonetheless, phosphorus in urban, agricultural, and industrial effluents is a major contributor to eutrophication, and removing it from these effluents is becoming a major concern. Phosphates are substances that include the element phosphorous, and they have an impact on water quality by encouraging algae to proliferate excessively, destroying other living forms, and producing dangerous poisons. Phosphate can promote their growth and play a key role in algal bloom outbreaks in eutrophic lakes and marines^2^. Keasling et al*.*^3^ found that the energy state of the cell and external phosphate levels regulate polyphosphate synthesis and breakdown. Under phosphate limiting-conditions, phosphate can be recovered from polyphosphate by hydrolysis. In all living cells, phosphorus plays an important role in the transport of metabolic energy and as a structural component of nucleotide and phospholipid molecules. Phosphorus deprivation results in an increase in cell size and form, as well as a thickening of the cell wall^4^. According to research^5^ studied the effects of phosphorus deficiency on the morphology, internal structure, and energy metabolic processes of the unicellular green alga *Scenedesmus obtusiusculus*, the number and size of lipid and starch globules also grow, and the internal organization of the cells gradually becomes chaotic. Data also showed that the cells' survival strategy in phosphorus-poor environments includes morphological and physiological adjustments that make it easier for the cells to move to and adapt to environments with a more favorable supply of phosphorus, like the frequently oxygen-poor but phosphorus-rich bottom zones.

Reference^6^ investigated phosphorus-limitation in the algae *Selenastrum minutum* and discovered that, in response to phosphorus limiting conditions, the rate of respiration inhibited five times, photosynthetic rates of carbon dioxide fixation and oxygen evolution inhibited by three times compared to control (phosphorus-containing culture), the rate of dark carbon dioxide fixation stimulated three times more than control, and the activities of phosphoenol pyruvate (PEP) carboxylase and PEP phosphatase significantly increased than control culture.

The effects of phosphorus deficiency on growth, photosynthesis activities, and some metabolic processes in the unicellular green alga *Chlorella kessleri* were studied by^7^ who proved that phosphorus-limiting conditions causes an increase in dry weight and a significant decrease in optical density, chlorophyll concentration, oxygen evolution, and dark respiration.

The active transport of metals into the cell appears to require phosphate^8^. Phosphate-limitation should thus directly reduce metal uptake, as has been observed in studies of nickel uptake by *Phaeodactylum tricornutum*^9^. In contrast, there is also conflicting data indicating an inverse relationship between phosphate and metals in the external medium. Metals may be complexed or precipitated by phosphate, rendering both harmful metals and phosphate unavailable to cells. If the metal concentration is high and phosphate is low, as indicated by^10^ growth effects of the metal may be compounded by the effects of phosphate deficiency. Excess metal is complexed when the phosphate content is raised, lowering toxicity while the surplus phosphate stimulates growth.

Planktonic algae are vital to aquatic communities because they are the foundation of many aquatic food chains. Nickel has been shown to concentrate in algae and hinder growth; nevertheless, the free nickel ion is the more harmful metal species to algae^11^.

Different *Dunaliella spp.* have a high protein quality that are considered a good nutritional source for humans, aquaculture, poultry, and animals^12^. *Dunaliella tertiolecta* was selected for this investigation for a variety of reasons. Extensive studies have been conducted on the physiology, biochemistry, ecology, molecular biology, and commercial applications of *Dunaliella* species, which are considered the main varieties that have been successfully employed to produce large concentrations of important compounds like lipids, proteins, carbs, and pigments.

### Aim of the work

The study was designed to assess the effect of phosphorous-limiting conditions in the absence and presence of different concentrations of nickel on growth, pigment content, photosynthesis, and respiration activities of the studied alga *Dunaliella tertiolecta*.

## Materials and methods

The biological material (unicellular sea green alga) *Dunaliella tertiolecta* 999 was obtained from the University of Texas at Austin's UTEX-alga culture collection. Algal culture was checked for axenia by inoculating it on Muller Hinton (M.H.) solid media^13^ described in Table 1, (S, Supplementary Table 1).

**Table 1:** Effect of different concentrations of dissolved nickel (mg/L) on growth measured as optical density of phosphorus supplemented and starved cells of *Dunaliella tertiolecta*.

Time (days)	Control		5	10	15	20	25	F (p)	LSD
4	** + P**	**0.33 ± 0.03**^**a**^	**0.330 ± 0.03**^**a**^	**0.315 ± 0.02**^**ab**^	**0.380 ± 0.04**^**abc**^	**0.245 ± 0.04**^**bc**^	**0.235 ± 0.03**^**c**^	5.096** (0.010)	0.076
	− P	0.30 ± 0.03^a^	0.34 ± 0.04^a^	0.32 ± 0.04^a^	0.29 ± 0.03^a^	0.21 ± 0.05^b^	0.15 ± 0.02^b^	13.530** (< 0.001)	0.050
8	** + P**	**0.60 ± 0.06**	**1.315 ± 0.07**^**a**^	**1.125 ± 0.08**^**ab**^	**0.753 ± 0.06**^**bc**^	**0.753 ± 0.07**^**c**^	**0.520 ± 0.03**^**d**^	17.330** (< 0.001)	0.083
	− P	0.54 ± 0.05^a^	0.57 ± 0.08^a^	0.54 ± 0.07^a^	0.42 ± 0.05^b^	0.36 ± 0.06^bc^	0.27 ± 0.07^c^	10.580** (< 0.001)	0.093
12	** + P**	**0.77 ± 0.06**^**a**^	**2.110 ± 0.09**^**a**^	**1.925 ± 0.14**^**ab**^	**1.513 ± 0.07**^**b**^	**1.230 ± 0.09**^**c**^	**0.440 ± 0.09**^**c**^	9.127** (0.001)	0.149
	− P	0.72 ± 0.09^ab^	0.79 ± 0.16^a^	0.70 ± 0.07^ab^	0.60 ± 0.08^bc^	0.50 ± 0.08^c^	0.30 ± 0.11^d^	9.127**(0.001)	0.149
16	** + P**	**1.03 ± 0.13**^**a**^	**2.495 ± 0.15**^**a**^	**2.488 ± 0.11**^**a**^	**1.788 ± 0.13**^**b**^	**1.063 ± 0.09**^**c**^	**0.355 ± 0.10**^**c**^	24.491** (< 0.001)	0.170
	− P	0.96 ± 0.08^a^	0.98 ± 0.17^a^	0.92 ± 0.20^a^	0.64 ± 0.10^b^	0.44 ± 0.12^bc^	0.30 ± 0.11^c^	12.794** (< 0.001)	0.200

*F F* test (ANOVA), Least significance difference at 0.05.

*Statistically significant at p ≤ 0.05.

**Statistically significant at p ≤ 0.001.

Different superscripts are significant.

Nickel was chosen for this study because of its presence in waste water, particularly in industrial locations, and because it has a potential risk when discharged into aquatic bodies^14^. A preliminary experiment was conducted following the methods of^15^ utilizing a wide range of varied concentrations of metal solutions [NiCl_2_] to establish the appropriate amounts of these metal salts that the examined alga could tolerate while avoiding direct deadly effects. The EC_50_ was around 15 mg/L, and the other four values were chosen (5, 10 mg/L, and 20, 25 mg/L). The growth of the investigated alga was determined by optical density and total chlorophylls.

In this investigation Atomic Absorption Spectrophotometer (Perkin-Elmer model 2380) equipped with an air-acetylene burner and HGA-400 graphite furnace was used to detect the different concentrations of nickel, and it was analyzed using hollow cathode lamps specific for Ni^2+^. For investigation of different concentrations of metal absorbed by algal cells within the experiment duration, 10 ml of algal suspension from all cultures was centrifuged at 5000 rpm for 30 min. the supernatants were taken for metal absorbed determination using Atomic Absorption Spectrophotometer. Cell pellets were suspended in 10 ml 0.1 M thiourea for 15 min then centrifuged and pellets were discarded and supernatants taken for adsorbed metal determination using Atomic Absorption Spectrophotometer as previously mentioned.

### Determination of optical density

Measurements of optical density (turbidity technique) are the most acceptable of all growth indices for determining growth rate. The change in percentage of light transmittance (T) relative to uninoculated control tubes set at 100% T was measured using a Perkin Elmer (Lambada 1) ultra violet spectrophotometer at 450 nm. The optical density was estimated using the equation of^16^

$${\text{Optical density}}\left( {{\text{O.D.}}} \right) = \log \left( {\frac{{I_{o} }}{I}} \right)$$

where: I = the transmittance of sample. I_O_ = the transmittance of blank adjusted to read 100%.

### Chlorophylls estimation

The simplest method for determining chlorophyll “a” and “b” concentrations is the spectrophotometer method. From the algal pellets obtained by centrifugation of 10 ml culture at 10,000 rpm for 20 min chlorophylls and β-carotene contents were extracted by using 3 ml 90% acetone. The pellets were kept in the dark, soaked in acetone, and stored in a refrigerator for 24 h. The acetone extract was centrifuged once more, and the supernatant was decanted for pigment estimations while, the remaining colourless pellets were discarded. This extract was made to a known volume and then transferred into the spectrophotometer cuvette. The absorption of chlorophyll pigments was measured at wave length of 647 nm and 664 nm. Taking the dilution into consideration, the concentration of chlorophylls “a” and “b” was estimated according to the trichromatic equation of^17^:

$${\text{Chlorophyll\,``a''}}\,\left( {{\text{mg}}.{\text{l}}^{ - 1} } \right)\, = \,11.93\,{\text{E}}_{664} \, - \,1.93\,{\text{E}}_{647}$$

$${\text{Chlorophyll\,``b''}}\,\,\left( {{\text{mg}}.{\text{l}}^{ - 1} } \right)\, = \,20.36\,{\text{E}}_{647} \, - \,5.50\,{\text{E}}_{664}$$

### Effect of phosphorus starvation

In order to study the relation between phosphorus limiting conditions and nickel toxicity, cells of *Dunaliella tertiolecta* were cultured in MH medium in the presence and absence of phosphorus (i.e., by removing KH_2_PO_4_ from the MH medium). 20 ml aliquots from the growing cells (as inoculums) were transferred to 250 ml culture flask containing 150 ml of MH medium free from phosphorus in the presence of the different concentrations of nickel already used. The cultures were incubated (at 25 °C and light intensity of 4000 Lux) in a controlled culturing chamber. Every 4 days and until the 16th day of culturing, the phosphorus starved cultures as well as those containing phosphorus were centrifuged (by centrifugation at 5000 g for 5 min), and the pellets obtained were used for the analysis of growth, chlorophyll content, O_2_ evolution, and O_2_ uptake.

The photosynthetic activity (O_2−_ evolution) was measured polarographically as oxygen evolution using a Clark type electrode (YSI, model 53). The actinic white light was obtained from a 150 W tungsten lamp The algae were harvested at 4 days interval until the end of the experiment (16 days).Cells separated from the medium (by centrifugation at 5000 × *g* for 5 min), were resuspended in 2 ml of 25 mM HEPES-KOH (pH 8.2) and transferred into an electrode chamber. Measurements were carried out using 3 ml of the algal suspension at room temperature (25 °C).Following consumption of all accessible inorganic carbon by the cells, the bicarbonate was added to the concentration of 50 or 150 μM and the rate of O_2_ evolution was measured in dark as O_2_- uptake in the same sample. The rate of photosynthetic O_2_ evolution was measured under a photon flux density of 500 μmol m^−2^ s^−1^.

### Statistical analysis

The gathered data were statistically examined using a two-way ANOVA (Analysis of Variance). Duncan's New Multiple Range Test (P 0.05) was used to examine the difference between the mean probability values. *F* tests were also used to determine the LSD (least significant difference), which was set at 0.05.

### Consent to participate

The author voluntarily agree to participate in this research study. I understand that even if I agree to participate now, I can withdraw at any time or refuse to answer any question without any consequences of any kind.

## Result and discussion

### Effect of phosphorus starved cultures of *Dunaliella tertiolecta* on growth represented as optical density under stress of nickel ions

In the case of normal culture, phosphorus starved control culture (without nickel stress), and phosphorus-starved treated cultures, data presented in Table 1 and graphed in figure (S1, Supplementary Data) clearly showed a progressive increase in optical density with increasing culturing period in case of normal culture, phosphorus-starved control culture, and phosphorus-starved treated cultures. Our findings are consistent with those of^18^ who found that in phosphorus starved cultures of three algae species, *Microcystic aeruginosa, Chlorella pyrenoidesa*, and *Cyclotella sp*., the biomass, specific growth rate, and Chl-a all declined significantly.

The optical density achieved during the four periods of culturing was lower in phosphorus-depleted control cultures than in normal cultures (i.e., cultures contained phosphorus). When compared to a normal control (without nickel addition), the optical density was reduced by 9.1% after 4 days of culturing under phosphorus deprivation and by 10.0 percent after 8 days of culturing. In the case of 5 mg/L dissolved nickel, however, the obtained optical density values in phosphorus starved treatment cultures rose with the increase in culturing period during all culturing periods as compared to phosphorus-starved control (without nickel addition) cultures.

At 10 mg/L dissolved nickel and after 4 days of culturing, the optical density although less than those in case of concentration 5 mg/L, yet it was higher than control (− P) but by increasing the culturing period more than 4 days, the optical density was less than control (− P). Our results are similar to those of^19^ who observed that the decrease in cell division rate signaled the onset of P-deficiency. The cultures that showed no significant increase in cell number for at least three consecutive days under the experimental conditions were considered P-depleted. In addition^20^, observed that the growth rate of *Dunaliella prava* was found to be dramatically lowered when phosphorus was limited. The content of chlorophyll fractions, total soluble carbohydrates, and proteins all fell considerably as a result of phosphorus restriction.

The results concerning the effect of dissolved nickel on the growth of *Dunaliella tertiolecta* under conditions of phosphorus limitation show that phosphorus starved *Dunaliella* had lower growth as compared to the control (phosphorus-containing culture medium). These results are in agreement with those obtained by^7^ who reported that the optical density of *Chlorella kessleri* cell suspension decreased with phosphorus deficiency compared to control. Also^21^, found that *Chlorella vulgaris* cells grew 30–40% slower in phosphorus-starved cultures than in control cultures. Furthermore^22^, showed that diatoms were unable to thrive when phosphorus levels were insufficient. Diatom dominances were reduced to 45 and 55% in enclosures where phosphate was not provided^23^ observed that, under salt stress, *Chlorella*'s metabolic rate was substantially lower than *Dunaliella*’s.

It can be concluded that when microorganisms are deprived of phosphorus, dissolved nickel uptake decreases, resulting in an increase in algal metabolism^24^. Also^25^, examined the effects of phosphorus and nitrogen starvation on the life cycle of *Emiliania huxleyi* (Haptophyta) and proved that various biochemical pathways’ metabolic load increased under P-starvation while it decreased under N-starvation.

### Effect of phosphorus starved cultures of *Dunaliella tertiolecta* on chlorophylls content under stress of nickel ions

Table 2 and figure (S2, Supplementary Data) show the sequences of change in the amount of chlorophylls a and b in phosphorus-depleted cultures of Dunaliella tertiolecta in response to various dissolved nickel concentrations. The results show that total chlorophyll content rose steadily until the end of the experiment under normal conditions (a control containing phosphorus). These results are in harmony with those obtained by^24^. The ratio between chlorophylls “a” and “b” remained nearly constant till the end of the 12th day. At the 16th day of culturing, the ratio decreased from 2.9:1 to 2.4:1. On the contrary, the total chlorophylls under control (in the absence of nickel element) in case of phosphorus-starved cultures showed a progressive increase up to the 12th day. At the 12th day the total chlorophylls in case of phosphorus-starved cultures decreased by 10.7% compared to the normal control. At the 16th day, the total chlorophylls in case of untreated phosphorus starved culture decreased by 20.8% compared to those obtained at normal control^26^. Reported that the chlorophyll content of Chlorella sorokiniana was significantly reduced due to a lack of nitrogen and phosphorus in the medium.

**Table 2:** Effect of different concentrations of dissolved nickel (mg/L) on chlorophylls content (µg/ml) of *Dunaliella tertiolecta* under the stress of phosphorus starvation.

**Day No**	Chlorophylls	Control + P	Control-P	5 mg/L	10 mg/L	15 mg/L	20 mg/L	25 mg/L
**4**	Chl.a	0.65	0.62	0.67	0.64	0.53	0.45	0.43
	Chl. b	0.21	0.20	0.21	0.21	0.18	0.18	0.19
	Total chl	0.86	0.82	0.88	0.85	0.71	0.63	0.62
	Ratio a/b	3.1:1	3.1:1	3.2:1	3.1:1	2.9:1	2.5:1	2.3:1
**8**	Chl.a	1.18	1.16	1.25	1.14	1.03	0.55	0.41
	Chl. b	0.41	0.41	0.43	0.39	0.36	0.29	0.21
	Total chl	1.68	1.57	1.68	1.50	1.39	0.84	0.62
	Ratio a/b	2.9:1	2.8:1	2.9:1	2.8:1	2.7:1	1.9:1	1.9:1
**12**	Chl. a	1.52	1.32	1.54	1.21	1.01	0.82	0.39
	Chl. b	0.53	0.51	0.57	0.46	0.39	0.32	0.22
	Total chl	2.05	1.83	2.11	1.67	1.40	1.14	0.61
	Ratio a/b	2.9:1	2.6:1	2.7:1	2.6:1	2.6:1	2.6:1	1.8:1
**16**	Chl.a	1.52	1.36	1.48	1.28	1.13	0.61	0.37
	Chl. b	0.64	0.62	0.59	0.58	0.54	0.30	0.23
	Total chl	2.16	1.71	2.07	1.86	1.67	0.91	0.60
	Ratio a/b	2.4:1	2.2:1	2.5:1	2.2:1	2.1:1	2.0:1	1.6:1

The total chlorophyll content of *Dunaliella tertiolecta* in the phosphorus-starved cultures treated with 5 mg/L of dissolved nickel increased gradually until the 12th day, when the content of the total chlorophylls reached 2.11 µg/ml, i.e., higher than the phosphorus-starved control (− P) by 15.3%. At the 16th day, the total chlorophylls, although lower than those obtained at the 12th day, were still higher than the control (− P). At a concentration of 10 mg/L of dissolved nickel, slight increase in the content of total chlorophylls was recorded from the beginning to the end of the culturing period, i.e., from the 4th to the 16th day. At the other concentrations of dissolved nickel (15, 20, and 25 mg/L), a pronounced decrease in the total chlorophylls could be observed from the 4th to the 16th day of culturing compared to control (− P). Our results are going with an agreement with those obtained by^27^ who found that chlorophylls were inhibited maximum at higher dissolved nickel concentrations but activated at lower values. The normal ratio between chlorophylls “a” and “b” (3:1) was upset after the 8^th^ day of culturing under concentrations 5, 10, and 15 mg/L of dissolved nickel. At 20 and 25 mg/L of dissolved nickel, this ratio was unstable from the beginning to the end of the experiment. The fact that dissolved nickel is extremely mobile and hence only absorbed to a minimal level may explain the sensitivity of the tested alga to nickel in response to phosphorus deficiency, and an increase in phosphorus concentration favors its absorption by microorganisms^28^. It can be concluded that when microorganisms are deprived of phosphorus, dissolved nickel uptake decreases, resulting in an increase in algal metabolism.

### Effect of different concentrations of dissolved nickel on photosynthesis (O_2_-evolution) of phosphorus starved cells of *Dunaliella tertiolecta*

Data represented in Table 3 and graphed in figure (S3, Supplementary Data S3) showed that the effect of phosphorus limitation on the photosynthetic activity of *Dunaliella tertiolecta* in response to five different concentrations of dissolved nickel revealed that, under phosphorus limiting conditions, the amount of O_2-_evolution was lower than in untreated cultures (the control). The evolution of O_2_ after 4 days of culturing in case of phosphorus starved control decreased by 8.7% compared to normal control, while after 12 days it decreased by 30.4%. The rate of O_2_-evolution at different concentrations of dissolved nickel over 5 mg/L caused successive reductions in the O_2_-evolution of phosphorus starved cells. Application of 5 mg/L of dissolved nickel, the results cleared that the rate of O_2_-evolution increased under the effect of all tested concentrations till the end of the experiment. It is clear from our data that the rate of O_2_-evolution depended mainly on the concentration of the nickel element and the length of culturing period. The lower the rate of O_2-_evolution, the higher the element's concentration, and the longer the culturing period. This coincided with the findings of^7^ who found that low phosphorus treatment causes *Chlorella kessleri* to lose its photosynthetic activity. In this regard, it was discovered that phosphorus deficiency resulted in a decrease in photosynthetic electron transport activity^29^ found that the O_2_-evolution of *Chlamydomon reinhardtii* declined by 75%. This decrease reflects damage of PSII and the generation of PSII Q_B_-non reducing centers.

**Table 3:** Effect of different concentrations of dissolved nickel (mg/L) on photosynthetic activity (O_2_-evolution calculated as µ mol O_2_ mg chl^-1^ h^-1^) on phosphorus supplemented and starved cells of *Dunaliella tertiolecta*.

Time (days)	Control		5	10	15	20	25	F (p)	LSD
4	** + P**	**473.00 ± 15.72**	**473.00 ± 13.86**^**b**^	**385.00 ± 11.36**^**c**^	**342.00 ± 11.53**^**d**^	**240.00 ± 7.94**^**e**^	**165.00 ± 8.19**^**f**^	324.296** **(< 0.001)**	**16.321**
	− P	432.00 ± 13.08^a^	471.00 ± 15.62^b^	406.00 ± 9.54^c^	371.00 ± 11.36^d^	264.00 ± 7.94^e^	182.00 ± 7.81^f^	303.459** (< 0.001)	16.365
8	** + P**	**352.00 ± 9.54**	**355.00 ± 10.58**^**b**^	**256.00 ± 7.94**^**c**^	**230.00 ± 7.81**^**d**^	**145.00 ± 7.94**^**e**^	**130.00 ± 7.81**^**f**^	324.739** **(< 0.001)**	**12.217**
	− P	322.00 ± 7.94^a^	342.00 ± 11.53^b^	301.00 ± 7.81^c^	251.67 ± 8.14^d^	153.00 ± 8.19^e^	135.00 ± 7.00^f^	318.609** (< 0.001)	12.450
12	** + P**	**316.00 ± 7.21**	**230.00 ± 7.00**^**b**^	**187.00 ± 7.81**^**c**^	**182.00 ± 8.66**^**c**^	**152.00 ± 8.89**^**d**^	**114.00 ± 7.00**^**e**^	208.694** **(< 0.001)**	**11.579**
	− P	220.00 ± 8.19^a^	232.00 ± 7.81^b^	203.00 ± 7.00^c^	193.00 ± 9.64 ^a^	170.00 ± 11.53^d^	123.00 ± 6.08^e^	161.307** (< 0.001)	12.460
16	** + P**	**170.00 ± 5.68**	**162.00 ± 5.57**^**a**^	**143.00 ± 6.93**^**b**^	**132.00 ± 6.25**^**c**^	**115.00 ± 7.21 **^**d**^	**102.00 ± 3.61**^**e**^	44.469** **(< 0.001)**	**9.279**
	− P	162.00 ± 7.81^a^	171.00 ± 7.00^a^	160.00 ± 9.64^a^	145.00 ± 7.21^b^	142.00 ± 7.94^b^	103.00 ± 3.46^c^	32.016** (< 0.001)	10.791

*F F* test (ANOVA).

Least significance difference at 0.05.

*Statistically significant at p ≤ 0.05.

**Statistically significant at p ≤ 0.001.

Different superscripts are significant.

Also^30^ found that P- deficiency has been correlated with lower photosynthetic rates. In the case of the treated phosphorus-starved cultures with lower concentrations (5 mg/L) of dissolved nickel, the rate of photosynthesis increased when compared to the phosphorus-starved control, but was less than that of the normal control (without nickel treatment). On the contrary, it was found that, in the treated phosphorus-starved cultures at concentrations of 10, 15, 20 and 25 mg/L of the tested element, the rate of photosynthesis decreased from the beginning to the end of the experiment. With increasing concentration, duration of the culturing period, and kind of element, the condition of decrease in O_2-_evolution became more pronounced; the same results were also recorded by^24^. The stimulation of growth and photosynthesis in the presence of some concentrations of dissolved nickel under phosphorus-limiting conditions is observed by^31^ they report that in Cu^2+^ sensitive *Scenedesmus acutus*, intracellular polyphosphate plays a key role in shielding photosynthesis from Cu^2+^ toxicity but not in copper resistant species.

### Effect of different concentrations of dissolved nickel on respiration (O_2-_uptake) of phosphorus starved cells of Dunaliella tertiolecta

Data obtained in Table 4 and graphed in figure (S4, Supplementary Data S4) concerning the rate of respiration of *Dunaliella tertiolecta* under phosphorus-limiting conditions was higher than that of untreated phosphorus-starved (control) for a short period of time only, i.e., after 4 days, at concentrations 5, 10 and 15 mg/L of dissolved nickel, After 8 days of culturing, the rate of O_2_- uptake increased only at 5 mg/L of dissolved nickel, while at the other concentrations it decreased gradually with increasing the concentration of the element. This finding is consistent with the findings of^23^, who discovered that *Dunaliella* cells increased their O_2_ absorption and evolution rates in the presence of 2 M salt NaCl in the media. In terms of oxygen uptake rate, *Dunaliella* cells demonstrated an increase in salt concentrations. In 1.5 M NaCl, it increased significantly by 60–80%.

**Table 4:** Effect of different concentrations of dissolved nickel (mg/L) on respiration activity (O_2_-uptake calculated as µ mol O_2_ h^-1^) on phosphorus supplemented and starved cells of *Dunaliella tertiolecta*.

Time (days)	Control		5	10	15	20	25	F (p)	LSD
4	** + P**	**1.67 ± 0.11**	**1.45 ± 0.11**^**a**^	**1.41 ± 0.09**^**a**^	**1.24 ± 0.08**^**b**^	**1.02 ± 0.09**^**c**^	**0.54 ± 0.06**^**d**^	47.232** **(< 0.001)**	**0.125**
	− P	1.32 ± 0.08^a^	1.63 ± 0.11^b^	1.47 ± 0.11^ab^	1.45 ± 0.10^a^	1.30 ± 0.11^a^	0.65 ± 0.08^c^	35.581** (< 0.001)	0.144
8	** + P**	**1.93 ± 0.05**^**a**^	**1.64 ± 0.13**^**a**^	**1.64 ± 0.16**^**a**^	**1.22 ± 0.08**^**b**^	**0.54 ± 0.03**^**c**^	**0.51 ± 0.09**^**c**^	70.705** **(< 0.001)**	**0.162**
	− P	1.61 ± 0.15^b^	1.83 ± 0.15^b^	1.74 ± 0.12^ab^	1.45 ± 0.12^c^	0.62 ± 0.05^d^	0.62 ± 0.10^d^	73.309** (< 0.001)	0.168
12	** + P**	**1.71 ± 0.10**^**a**^	**1.51 ± 0.15**^**a**^	**1.35 ± 0.15**^**a**^	**1.01 ± 0.09**^**b**^	**0.51 ± 0.06**^**c**^	**0.34 ± 0.03**^**c**^	40.390** **(< 0.001)**	**0.202**
	− P	1.50 ± 0.14^a^	1.68 ± 0.14^a^	1.46 ± 0.13^a^	1.04 ± 0.10^b^	0.60 ± 0.08^c^	0.54 ± 0.08^c^	36.065** (< 0.001)	0.204
16	** + P**	**1.60 ± 0.12**	**1.40 ± 0.11**^**a**^	**1.32 ± 0.05**^**a**^	**0.96 ± 0.08**^**b**^	**0.42 ± 0.06**^**c**^	**0.32 ± 0.07**^**c**^	47.183** **(< 0.001)**	**0.147**
	− P	1.40 ± 0.13^a^	1.43 ± 0.13^a^	1.35 ± 0.06^a^	1.02 ± 0.11^b^	0.56 ± 0.10^c^	0.43 ± 0.04^c^	47.183** (< 0.001)	0.162

*F F* test (ANOVA), *LSD* least significance difference at 0.05.

*Statistically significant at p ≤ 0.05.

**Statistically significant at p ≤ 0.001.

Different superscripts are significant.

Concerning the increase in respiration in P-depleted green alga species cultures^5^ suggested that *Scenedesmus*, for example, can utilize the energy stored in starch and lipids for active phosphorus uptake from lake sediments. This process is aided by an increase in phosphatase production^32^ and these cells' ability to operate anaerobically^33^. When unicellular green algae or higher plants are exposed to P deficiency, the majority of newly fixed carbon appears to be allocated to the synthesis of non-phosphorylated storage polyglucans (i.e., starch) or sucrose, with less photosynthetic activity directed to respiratory metabolism and other biosynthesis pathways^34^. It can be concluded from the obtained results that, when the alga was cultivated under phosphorus deficiency and treated with varied amounts of dissolved nickel, the growth was the most sensitive characteristic, followed by photosynthesis, and then dark respiration. In the few comparative studies with several species of green algae, growth was more sensitive than the other physiological processes examined. Out of them^35^, reported that growth was more susceptible to phosphorus deficiency in *Chlorella pyrenoidosa* and *Asterionella gracilis* than photosynthesis and respiration (the least sensitive processes). Growth was also more sensitive than photosynthesis in *Nitzschia closterium* ^36^ . Another important fact reported by^37^ is that under low phosphorus conditions, *Dunaliella parva* accumulates lipids rather than carbohydrates*.* These findings imply that phosphorus stress may prevent starch and/or protein production, leading to an increase in carbon flux to lipids.

## Conclusion

Concerning the effect of different concentrations of dissolved nickel on the growth of *Dunaliella tertiolecta* under conditions of phosphorus limitation, the results showed that phosphorus-starved cells had lower growth and total chlorophyll contents compared to control (without nickel treatment), depending on metal concentration and length of the culturing period. As for the effect of phosphorus-limiting conditions on the photosynthetic activity of *Dunaliella tertiolecta* cells in response to the five different concentrations of dissolved nickel, the results obtained showed that the amount of O2-evolution with phosphorus limiting conditions was lower than of untreated normal cultures (control + P). The rate of photosynthesis at the treated phosphorus starved cultures at lower concentrations (5 mg/L) of dissolved nickel increased when compared to the phosphorus-starved control (without nickel treatment), but was less than that at in the normal control. On the contrary, in the treated phosphorus starved cultures at concentrations of 10, 15, 20, and 25 mg/L, the rate of photosynthesis decreased from the beginning to the end of the experiment.

## Supplementary Information

Supplementary Figures.

Supplementary Table 1.

## Data Availability

All data generated or analyzed during this study are included and available in this article.
