# Supplementary material for: Algal sensitivity to nickel toxicity in response to phosphorus starvation
Source: Sci Rep. 2022 Dec 5;12:21033. doi: 10.1038/s41598-022-25329-5 (PMC9722719; doi:10.1038/s41598-022-25329-5)
Supplement: Supplementary file 1 — Supplementary Figures. [file 41598_2022_25329_MOESM1_ESM.docx]

**Figure (1 +P &-P): Effect of different concentrations of** **dissolved nickel (mg/L) on growth measured as optical density of phosphorus supplemented (+P) and starved cells (-P) of *Dunaliella tertiolecta*.**

**Figure (2): Effect of different concentrations of dissolved nickel (mg/L) on chlorophylls content (mg/L) of of phosphorus supplemented (+P) and starved cells (-P) of *Dunaliella tertiolecta***

**Figure (3 +P &-P): Effect of different concentrations of dissolved nickel (mg/L) on O_2_-evaluation (µmol O_2_ mg chl^-1^ h^-1^) of phosphorus supplemented (+P) and starved cells (-P) of *Dunaliella tertiolecta*.**

**Figure (4 +P &-P): Effect of different concentrations of dissolved nickel (mg/L) on O_2_-uptake (µmol O_2_ h^-1^) of phosphorus supplemented (+P) and starved cells (-P) of *Dunaliella tertiolecta*.**
