# Supplementary material for: Algal sensitivity to nickel toxicity in response to phosphorus starvation
Source: Sci Rep. 2022 Dec 5;12:21033. doi: 10.1038/s41598-022-25329-5 (PMC9722719; doi:10.1038/s41598-022-25329-5)
Supplement: Supplementary file 2 — Supplementary Table 1. [file 41598_2022_25329_MOESM2_ESM.docx]

**Table (1): Amount of salts per liter of MH medium:**

| **Salts** | **Amount per liter** |
| --- | --- |
| NaCl  MgCl_2_.6H_2_O  MgSO_4_.7H_2_O  KCl  CaCl_2_.2H_2_O  KNO_3_  NaHCO_3_  *KH_2_PO_4_  EDTA  FeCl_3_.6H_2_O  ZnCl_2_  H_3_BO_3_  CoCl_2_.2H_2_O  CuCl_2_.2H_2_O  MnCl_2_.4H_2_O  (NH_4_)_6_Mo_7_O_24_.4H_2_O | 73.050 g  1.500 g  0.500 g  0.200 g  0.200 g  1.000 g  0.043 g  0.035 g  1.890 mg  2.440 mg  0.041 mg  0.610 mg  0.015 mg  0.041 mg  0.410 mg  0.300 mg |

pH was adjusted at 7.5

*potassium phosphate solution was autoclaved separately and added aseptically to the sterilized medium to avoid phosphate precipitation. (Incubation was under controlled culturing chamber temperature at 25˚C ± 3˚C and light at 4000 lux).
